# *FIP1L1-PDGFRA *molecular analysis in the differential diagnosis of eosinophilia

**DOI:** 10.1186/1471-2326-9-1

**Published:** 2009-02-02

**Authors:** Gedeon Loules, Fani Kalala, Nikolaos Giannakoulas, Emmanouil Papadakis, Panagiota Matsouka, Matthaios Speletas

**Affiliations:** 1 Department of Immunology and Histocompatibility, University of Thessaly Medical School, University Hospital of Larissa, Larissa, Greece; 2 Department of Hematology, University of Thessaly Medical School, University Hospital of Larissa, Larissa, Greece; 3 Department of Hematology, Papageorgiou General Hospital, Thessaloniki, Greece

## Abstract

**Background:**

Primary eosinophlia associated with the *FIP1L1-PDGFRA *rearrangement represents a subset of chronic eosinophilic leukaemia (CEL) and affected patients are very sensitive to imatinib treatment. This study was undertaken in order to examine the prevalence and the associated clinicopathologic and genetic features of *FIP1L1-PDGFRA *rearrangement in a cohort of 15 adult patients presenting with profound eosinophilia (> 1.5 × 10^9^/L).

**Methods:**

Reverse transcriptase-polymerase chain reaction (RT-PCR) was used for the detection of *FIP1L1-PDGFRA *rearrangement and the results confirmed by direct sequencing. *C-KIT*-D816V mutation was analysed retrospectively by PCR and restriction-fragment-length-polymorphism (PCR-RFLP), in all cases with primary eosinophilia.

**Results:**

Two male patients with splenomegaly carried the *FIP1L1-PDGFRA *rearrangement, whilst 2 others were ultimately classified as suffering from idiopathic hypereosinophlic syndrome (HES) and one from systemic mastocytosis. These patients were negative for the *C-KIT*-D816V mutation and received imatinib (100–400 mg daily). Patients with CEL and HES responded to imatinib and remained in complete haematological, clinical and molecular (for carriers of *FIP1L1-PDGFRA *rearrangement) remission for a median of 28.2 months (range: 11–54), whilst the patient with systemic mastocytosis did not respond. Interestingly, in both patients with *FIP1L1-PDGFRA *rearrangement, the breakpoints into *PDGFRA *were located within exon 12 and fused with exons 8 and 8a of *FIP1L1*, respectively.

**Conclusion:**

An early diagnosis of *FIPIL1-PDGFRA*-positive CEL and imatinib treatment offer to the affected patients an excellent clinical therapeutic result, avoiding undesirable morbidity. Moreover, although the molecular mechanisms underlying disease pathogenesis remain to be determined, imatinib can be effective in patients with idiopathic HES.

## Background

Eosinophilia (> 0.5 × 10^9^/L) is a common clinical finding that can be secondary to a large variety of diseases. When evaluation of eosinophilia fails to reveal an underlying disease, the diagnosis of hypereosinophilic syndrome (HES) is evocated. HES is defined by (1) eosinophilia (> 1.5 × 10^9^/L) for more than 6 months; (2) exclusion of reactive eosinophilia caused by parasitic infections, allergies, or other known causes, as well as eosinophilia associated with neoplasias; and (3) evidence of end-organ damage [1-4]. Over the last decade, great progress has been made in understanding the molecular basis of HES that has resulted in the characterization of specific genetic alterations linked to clonal eosinophilia. The most frequent genetic aberration is the cryptic deletion of 4q12, i.e. del(4)(q12), producing the FIP1-like 1/platelet-derived growth factor receptor alfa (*FIP1L1-PDGFRA*) fusion transcript, which results in an eosinophilic, myeloproliferative disorder (chronic eosinophilic leukaemia, CEL) [5]. In addition, in a subset of patients with HES, eosinophilia is secondary to a primitive Th2 lymphoid disorder, overproducing interleukin-5 (IL-5), indicating the existence of lymphocyte-mediated HES [3].

The *FIP1L1-PDGFRA *fusion gene induces increased tyrosine kinase activity of PDGFRA [5,6], and is present in approximately 10–15% of patients with HES [5,7,8]. Despite the fact that these patients appear to have a more severe disease phenotype, involving extensive end-organ pathology [5,8-11], they are dramatically sensitive to treatment with the tyrosine kinase inhibitor imatinib [5,8-13]. Thus, early detection of *FIPIL1-PDGFRA *rearrangement and the subsequent imatinib administration can offer to the affected patients an excellent clinical therapeutic outcome, avoiding undesirable morbidity and mortality.

This study was undertaken in order to examine both the prevalence and the associated clinicopathologic and genetic features of *FIP1L1-PDGFRA *rearrangement in a cohort of 15 adult patients presenting with eosinophilia and an absolute eosinophil count higher than 1.5 × 10^9^/L.

## Methods

### Patients

Peripheral blood (PB) and/or bone marrow (BM) from 15 patients (male/female: 7/8, mean age: 45.2 years, range: 22–72 years) with eosinophilia (eosinophils > 1.5 × 10^9^/L), without an unambiguous history of allergic diseases, were referred to the Immunology Lab and examined for the presence of *FIP1L1-PDGFRA *rearrangement. Regardless of the molecular analysis, a complete clinical and laboratory examination was also performed. The diagnosis of idiopathic HES, systemic mastocytosis and/or CEL was based either on standard diagnostic criteria [1-3] or on the result of the molecular analysis. Cytogenetic analysis was performed in all, but one, patients with primary eosinophilia and in two patients, for which ultimately another diagnosis was made. Flow cytometric analysis for the demonstration of CD3-CD4+ or CD3+CD4-CD8- clones, implicated in lymphocyte-mediated HES, was performed in all patients, but serum vitamin B12, IL-5 and mast cell tryptase levels were not assayed. Detection of *C-KIT*-D816V mutation was performed retrospectively in all cases with primary eosinophilia.

Written informed consent was obtained from all patients and the study was approved by the Institutional Review Board of both hospitals (University Hospital of Larissa and Papageorgiou General Hospital, Greece).

### Molecular analyses

#### Detection of FIP1L1-PDGFRA fusion

The *FIP1L1-PDGFRA *rearrangement was analyzed by a modified nested reverse transcriptase-polymerase chain reaction (RT-PCR) protocol [14]. In particular, RNA was extracted from BM or PB, and cDNA was reversed transcribed, as previously described [15]. One sixth of the synthesized cDNA was used in a first PCR reaction of 30 μL, using 62.5 μM of each deoxynucleoside triphosphate, 20 pmol of each primer (sense: 5'-ACCTGGTGCTGATCTTTCTGAT-3' and antisense: 5'-TGAGAGCTTGTTTTTCACTGGA-3'), 1.6 mM MgCl_2 _and 1.4 μL of Taq Elongase (Invitrogen, UK) in a PCR buffer supplied by the manufacturer. Thereafter, 1 uL of the first PCR product was used as template for the nested PCR reaction (30 uL), along with 62.5 μM of each deoxynucleoside triphosphate, 10 pmol of each primer (sense: 5'-AAAGAGGATACGAATGGGACTTG-3' and antisense: 5'-GGGACCGGCTTAATCCATAG-3'), 1.5 mM MgCl_2 _and 0.5 U of Taq Polymerase (Invitrogen, UK) in a PCR buffer supplied by the manufacturer. The primary PCR conditions were: 2 min at 94°C followed by 32 cycles (94°C for 45 sec, 56°C for 45 sec, 68°C for 75 sec) followed by 5 min at 68°C. The conditions for the nested PCR were: 2 min at 94°C followed by 25 cycles (94°C for 30 sec, 60°C for 30 sec, 72°C for 60 sec) followed by 5 min at 72°C after the last cycle. All PCR amplifications were carried out in the PCR-engine apparatus PTC-200, MJ Research (Watertown-Mass., USA). The first and nested PCR products were analyzed in 2.5% TBE agarose gels. The cell line EOL-1 (with known *FIP1L1-PDGFRA *rearrangement) served as positive control in our experiments.

For the confirmation of nested PCR results, PCR products with suspected *FIP1L1-PDGFRA *rearrangement were purified by QIAquick gel extraction kit (Qiagen, UK) and directed sequenced using an ABI Prism 310 Genetic Analyzer (Applied Biosystems, Foster City, CA) and a Big Dye Terminator DNA sequencing kit (Applied Biosystems).

#### Mutational analysis of C-KIT

The *C-KIT*-D816V mutation was analysed at the genomic DNA level. In particular, DNA was extracted using the QIAamp DNA Blood Mini kit (Qiagen, UK), according to the manufacturer's instructions. Semi-nested PCR was performed, as previously described [16], with some modifications. In particular, the first PCR was performed in a final volume of 30 μL containing 100 uM of each dNTP, 1.5 mM MgCl_2_, and 0.5 U Taq polymerase (Invitrogen, UK), and 10 pmol of each primer (sense: 5'-CACAGAGACTTGGCAGCCAG-3' and antisense: 5'-CAGGATTTACATTATGAAAGTCACGG-3'). Aliquots of 1 μL of the PCR product served as template for the semi-nested PCR, using the same concentrations as above. The primers for the nested PCR were: sense 5'-ATCCTCCTTACTCATGGTCGGATC-3' and the same antisense used for the primary PCR. Each PCR cycle consisted of 30 seconds denaturation at 94°C, 30 sec annealing at 56°C, and 45 sec extension at 72°C. The first cycle was preceded by two minutes denaturation at 94°C. The last cycle was extended by a five minute elongation at 72°C. The first PCR run for 30 cycles and nested-PCR for 25 cycles. All PCR amplifications were carried out in the PCR-engine apparatus PTC-200, MJ Research (Watertown-Mass., USA).

To detect the presence of the *C-KIT*-D816V mutation, the semi-nested PCR products were digested with the endonuclease HinfI (New England Biolabs, USA) and the restriction products were analysed by electrophoresis on a 4% agarose TBE gels. The *C-KIT*-D816V mutation creates a second restriction site within the semi-nested PCR products. The predicted sizes for the wild-type sequence were 121 bp and 68 bp, and for the mutated sequence 121 bp, 68 bp, 54 bp, and 14 bp [16].

## Results

Amongst the 15 patients with eosinophilia, only two were positive for the *FIP1L1-PDGFRA *rearrangement (13,3%). Two others were classified as idiopathic HES and another as systemic mastocytosis, after bone marrow and lymph node biopsies, indicating mast cell infiltration. A summary of the clinical and laboratory findings for these patients is presented in Table 1.

**Table 1:** Clinical and laboratory findings of the patients with primary eosinophilia

No	Diagnosis	Sex	Age	Clinical presentation	WBC/eosinophils (×10^9^/L)	Hb (g/dl)	PLT (×10^9^/L)	Previous therapy	Imatinib dosage	Duration of imatinib therapy	Result of imatinib therapy
1	CEL	M	34	Maculopapular rash, fatigue, peripheral neuropathy, splenomegaly	20.4/11.4	12.5	210	-	400 mg (I) 100 mg (M)	35 mo	CHR, clinical, molecular remission

2	CEL	M	22	Splenomegaly	27.0/10.2	13.1	157	-	100 mg (I) 100 mg (M)	11 mo	CHR, clinical, molecular remission

3	Idiopathic HES	M	23	Abdominal pain, lymphadeno-pathy, fatigue, cough	36.1/16.2	13	310	Steroids for 6 mo	400 mg (I) 100 mg (M)	54 mo	CHR

4	Idiopathic HES	M	38	Cough, asthma	21.9/14.0	14.8	268	-	400 mg (I) 400 mg (M)	13 mo	CHR

5	SM-Eo	F	37	Malaise, arthralgia, gastritis, splenomagaly	13.8/5.5	12.7	673	IFN-a for 2 y	400 mg	12 mo	No response

Abbreviations: CEL, chronic eosinophilic leukaemia; HES, hypereosinophilic syndrome; SM-Eo, systemic mastocytosis with eosinophilia; M, male; F, female; WBC, white blood cell count; Hb, haemoglobin; PLT, platelets; I, induction therapy; M, maintenance therapy; CHR, complete haematological response.

Within the remaining patients, one suffered from eosinophilic gastroenteritis, two from asthma (all responded to steroids), two from atypical myeloproliferative syndrome (one was also positive to *JAK2*-V617F mutation and both responded to hydroxyurea therapy), one from dermatomyocytis, one from bronchiectasis, and one from chronic idiopathic urticaria. In two cases, the eosinophilia was not permanent and ascribed to possible allergic causes (also responded to steroids). Notably, only the patient with eosinophilic gastroenteritis displayed eosinophilia for more than 6 months.

All patients with primary eosinophilia (CEL, HES and systemic mastocytosis) were negative for the *C-KIT*-D816V mutation, while cytogenetic analysis in all, but one patient (case 2 of Table 1), due to dry-tap on bone marrow aspiration, was normal. Moreover, a clonal interleukin-5-producing T-cell population was not detected by flow cytometry in any of the above patients.

All patients with primary eosinophilia negative for the *FIP1L1-PDGFRA *rearrangement, displayed eosinophilia for more than 6 months (mean: 7 months, range: 6–8). Amongst them, one with idiopathic HES was receiving imatinib as first line therapy and the others were receiving imatinib after failure of their previous treatment (Table 1). The patients with idiopathic HES responded to imatinib in a dosage of 400 mg per day, displaying CHR and clinical remission, and continue on imatinib, till now (Table 1). The last patient, suffering from systemic mastocytosis with eosinophilia, was negative to *FIP1L1-PDGFRA *rearrangement, as well as to the *C-KIT*-D816V mutation, but did not respond to imatinib treatment. Interestingly, she had initially received IFN-alfa subcutaneously for 2 years, with no response, and then received imatinib (400 mg per day), for which she was also not sensitive.

**Figure 1:**
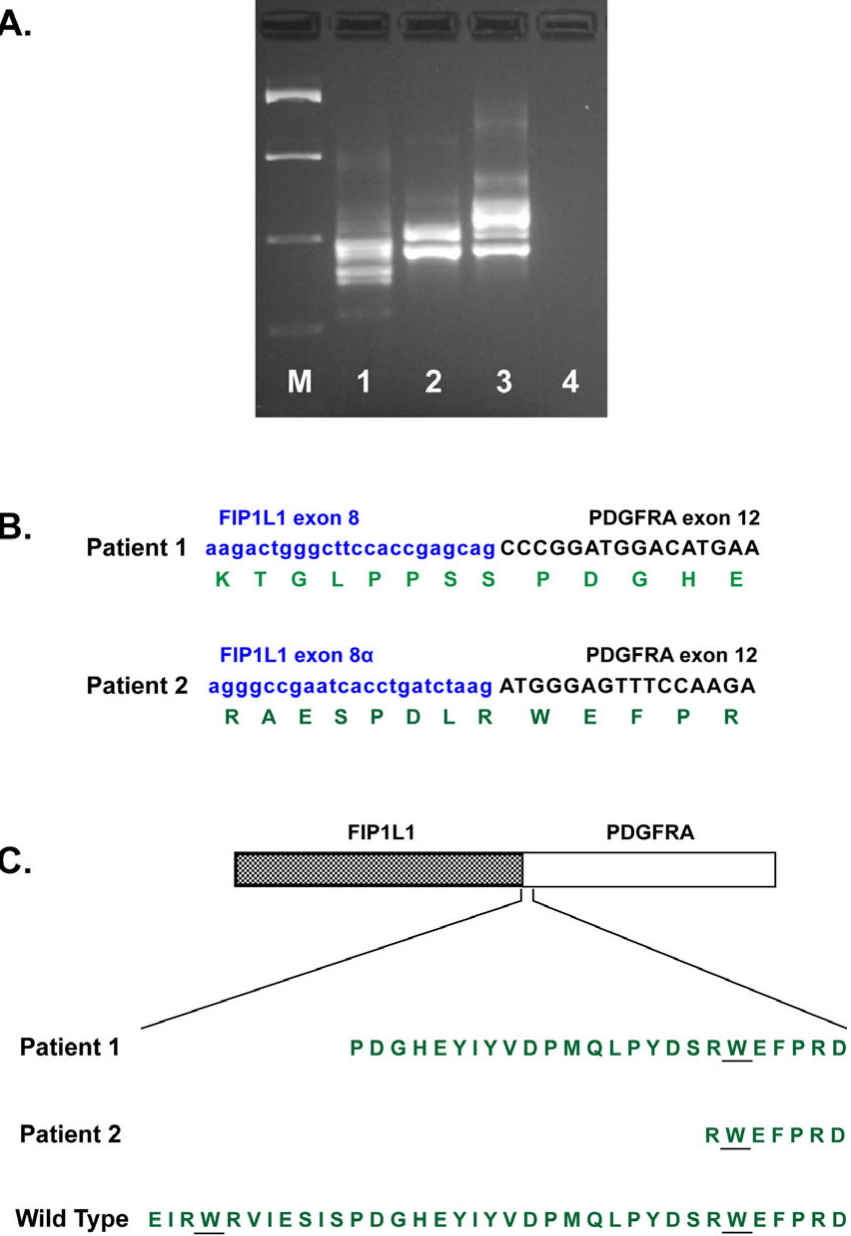
**Molecular analyses of patients with *FIP1L1-PDFGRA *rearrangement**. A. Reverse transcriptase-polymerase chain reaction (RT-PCR) analysis of the *FIP1L1-PDFGRA *fusion gene isolated from bone marrow and peripheral blood of patients with chronic eosinophilic leukaemia at diagnosis. M: 100 bp ladder molecular weight marker (Invitrogen, UK); Lane 1: case 1; Lane 2: case 2; Lane 3: cell line EOL-1 (positive control), Lane 4: negative PCR control (blank). It is noteworthy that both patients, as well as the cell line EOL-1 (positive control), display more than one mRNA isoforms of the fusion gene. B. Sequence variants for each patient with the fusion gene. *FIP1L1 *sequences are shown in lowercase and in blue, and *PDGFRA *sequences are shown in uppercase and in black. Exon numbering in *FIP1L1 *is based on a complementary DNA (cDNA) clone (GenBank accession number NM_030917). The amino acid sequence of the chimeric protein in site of fusion is indicated in green. C. Schematic representation of the FIP1L1-PDGFRA fusion protein. In both cases the breakpoints in *PDGFRA *are located within the juxtamembrane region, between the two tryptophan (W) residues of the putative WW-domain.

Considering the patients carrying the *FIP1L1-PDGFRA *rearrangement, they were males, exhibiting splenomagaly. Both patients displayed more than one isoforms of the fusion gene (Figure 1), similar to the majority of the affected patients, as described in the literature. The RT-PCR results were confirmed in both cases by sequencing analysis. Interestingly enough, as for all patients reported in the literature, the breakpoint into the *PDGFRA *gene was located in exon 12 (different for each patient), that was fused with exon 8 of the *FIP1L1 *gene for the first patient and exon 8a for the second (Figure 1). Moreover, the connection site between the genes was the same for the isoforms of each patient, and their different length was due to deletions in the *FIP1L1 *gene (for example in case 1 of Table 1, one isoform was characterized by deletion of the whole exon 7a of the *FIP1L1 *gene).

A more detailed clinical and laboratory presentation of patients carrying the *FIP1L1-PDGFRA *rearrangement is presented below:

### Case 1

The first patient presented maculo-papular rash on lower extremities and abdomen and an absolute eosinophil count of 2 × 10^9^/L, one and half year before admission to the hospital. Initially the rash was considered to be of allergic etiology and the patient received local corticosteroids by a dermatologist. Nevertheless, a progressive deterioration of the rash was observed, and the patient also complained of fatigue and muscle weakness accompanied by hypaesthesia of the peripheral parts of the lower extremities that led to the weakness of walk, one week prior to admission. At that time, the laboratory tests showed that the eosinophils increased to 11.4 × 10^9^/L and moreover, a mild anaemia (Hb: 12.5 gr/dl) was present. Clinical examination revealed splenomegaly (palpable spleen 8 cm below the left costal margin), vesicular exanthema on the abdomen and the low extremities and absent deep tendon reflexes. Bone marrow aspiration and biopsy revealed an extensive eosinophil infiltration (68%), whilst the cytogenetic analysis was normal. Molecular analysis excluded the presence of *BCR-ABL *transcript, while a *FIP1L1-PDGFRA *rearrangement was documented (Figure 1A). Imatinib was initiated at a dosage of 400 mg and after 14 days of treatment, the eosinophil count significantly declined along with a dramatic reduction of splenomegaly. Imatinib was well tolerated with an initial decrease of Hb levels to 9 gr/dl, that was resolved by reducing the imatinib dosage to 200 mg per day and administrating 150 mcg of darbepoetin alfa, twice weekly subcutaneously. The mobility and the hypaesthesia were recovered 2 months later and PB and BM molecular signal of the *FIP1L1-PDGFRA *fusion gene were undetectable 4 months after treatment. Six months later, the imatinib dosage further declined to 100 mg per day, and the patient is currently followed-up as an outpatient.

### Case 2

The second patient displayed eosinophilia (10.2 × 10^9^/L) and mild anaemia (Hb: 13.1 gr/dl) during a routine laboratory examination. A clinical examination revealed only splenomegaly (palpable spleen 2 cm below the left costal margin). Bone marrow aspiration was dry-tap, and for this reason cytogenetic analysis was not performed. However bone marrow biopsy revealed an extensive infiltration (80%) by eosinophils. Molecular analysis revealed the presence of the *FIP1L1-PDGFRA *rearrangement. Imatinib was started at a dosage of 100 mg two weeks after the estimation of eosinophilia and after 14 days of treatment, the eosinophil count become normal (0.3 × 10^9^/L) and the spleen was not palpable below the left costal margin. Imatinib was well tolerated without side effects, and PB and BM molecular signal of the *FIP1L1-PDGFRA *fusion gene was undetectable 3 months after treatment.

## Discussion

In this study we describe our experience for the diagnostic utility of the detection of *FIP1L1-PDGFRA *rearrangement in patients with profound eosinophilia. In our cohort, the prevalence of *FIP1L1-PDGFRA *rearrangement was 13.3% in the total analysed group and 40% among patients with primary eosinophilia. Till now, the recorded proportion of *FIP1L1-PDGFRA*-positive cases among such patients varies widely, ranging from 0% to 56%, probably reflecting small sample sizes, as in our cohort, and variable inclusion criteria [5,7-13,17]. In the study, with the largest cohort, Pardanani and co-workers screened 741 patients with moderate to severe eosinophilia and reported a 3% prevalence of *FIP1L1-PDGFRA *fusion positivity [12]. Moreover, Klion also noted that 10%–50% of patients meeting the classic definition of HES may be *FIP1L1-PDGFRA*-positive [18]. It is obvious, that a subsequent meta-analysis, taking into account all published studies, could clarify the true prevalence of *FIP1L1-PDGFRA *rearrangement in patients with idiopathic eosinophilia and HES.

The detection of *FIP1L1-PDGFRA *rearrangement in the second patient, directly after the estimation of eosinophilia, resulted in the early diagnosis of CEL, with a profound impact on the patient's clinical course and outcome. Thus, it is not unreasonable to speculate that the usage of molecular techniques for the early diagnosis of suspected *FIP1L1-PDGFRA*-positive leukemias, can modify the diagnostic criteria of hypereosinophilic syndrome with significant impact in undesirable morbidity and mortality.

Interestingly, in both patients with *FIP1L1-PDGFRA *rearrangement, the breakpoints into *PDGFRA *were located in exon 12 and fused with exons 8 and 8a of *FIP1L1*, respectively. Till now, all described breakpoints occurred in an *FIP1L1 *intron (spread from 7 to 10 at genomic DNA level) and in exon 12 of *PDGFRA *[5,9]. Although in all reported cases, the *PDGFRA *breakpoints were variable, they were limited to exon 12 and more specifically within the region encoding a WW-like domain [2]. A similar finding was also observed in our patients (Figure 1). There is strong evidence that the result of *FIP1L1-PDGFRA *rearrangement is an interrupted *PDGFRA *juxtamembrane region, due to an interstitial deletion of a tryptophan (W) residue of the putative WW-domain (Figure 1). This domain is believed to be a negative regulator of kinase activity and serves as an auto-inhibitory domain. Thus, the interruption of the juxtamembrane region of *PDGFRA *may serve as the primary mode of constitutive kinase activation and the leukemic transformation of the affected cells [2,19].

In our study, both patients with *FIP1L1-PDGFRA *rearrangement were male and exhibited splenomegaly, similar to the majority of the positive patients reported in the literature. It is noteworthy that one patient displayed rash as a presenting sign, considering that skin involvement is rare in CEL [11,20]. Moreover, this patient displayed undesirable morbidity due to initial misdiagnosis, while the second one was diagnosed early after the demonstration of eosinophilia in routine laboratory examination, without reference to the diagnostic criteria of HES [2-4]. This point should be highlighted, considering that even in such patients, cardiac damage can be irreversible. On the other hand, 2 HES patients were also sensitive to imatinib treatment, and remained in CHR, receiving imatinib 9 and 50 months, respectively, after diagnosis. It has been reported that up to 40% of imatinib responding patients lack the *FIP1L1-PDGFRA *fusion [5,11,17,20], suggesting the activation of other, still unknown, tyrosine kinases that may contribute to disease pathogenesis and phenotype. Although in a recent report, Baccarani and coworkers reported that such patients need higher doses of imatinib and usually relapse [11], this does not appear to be absolutely the rule [5,17], since in our cohort 2 HES patients remain in CHR and clinical remission, receiving 100 and 400 mg of imatinib, respectively, as maintenance treatment.

Moreover, we treated another patient with primary eosinophilia, whose diagnosis was ultimately systemic mastocytosis. Regardless of the presence or absence of *FIP1L1-PDGFRA *or *C-KIT*-D816V, patients with SM may present with eosinophilia [21]. It is well documented today that some of these patients carry the *FIP1L1-PDGFRA *rearrangement and are sensitive to imatinib treatment, suggesting that the molecular pathogenesis for this subset of patients is similar to that of CEL [14,21]. Indeed, it has been recently proposed that these patients should be appropriately classified as systemic mastocytosis-CEL [21]. However, our patient was negative for the *FIP1L1-PDGFRA *rearrangement, as well as for the *C-KIT*-D816V mutation, and did not respond to imatinib treatment.

## Conclusion

An early diagnosis of *FIP1L1-PDGFRA*-positive CEL and imatinib treatment can offer to affected patients an excellent clinical therapeutic result, avoiding undesirable morbidity and mortality. Moreover, although the molecular mechanisms underlying disease pathogenesis and phenotype remain to be determined, imatinib can also be effective in patients with idiopathic HES.

## Competing interests

The authors declare that they have no competing interests.

## Authors' contributions

MS was primarily responsible for this work, from conception to submission of the manuscript and wrote the paper. GL carried out the molecular genetic studies and helped drafting of the manuscript. FK carried out the flow cytometric analyses and contributed to the data interpretation. NG, EP and PM participated in study design and the collection and interpretation of data. All authors read and approved the final manuscript.

## Pre-publication history

The pre-publication history for this paper can be accessed here:


